# Pulmonary and Immune Dysfunction in Pediatric Long COVID: A Case Study Evaluating the Utility of ChatGPT-4 for Analyzing Scientific Articles

**DOI:** 10.3390/jcm14176011

**Published:** 2025-08-25

**Authors:** Susanna R. Var, Nicole Maeser, Jeffrey Blake, Elise Zahs, Nathan Deep, Zoey Vasilakos, Jennifer McKay, Sether Johnson, Phoebe Strell, Allison Chang, Holly Korthas, Venkatramana Krishna, Manojkumar Narayanan, Tuhinur Arju, Dilmareth E. Natera-Rodriguez, Alex Roman, Sam J. Schulz, Anala Shetty, Mayuresh Vernekar, Madison A. Waldron, Kennedy Person, Maxim Cheeran, Ling Li, Walter C. Low

**Affiliations:** 1 Department of Neurosurgery, University of Minnesota, Minneapolis, MN 55455, USA; setjohn8@gmail.com (S.J.); strel048@umn.edu (P.S.); dilma17n@umn.edu (D.E.N.-R.); shett098@umn.edu (A.S.); 2 Stem Cell Institute, University of Minnesota, Minneapolis, MN 55455, USA; 3 Bioinformatics and Computational Biology Graduate Program, University of Minnesota, Minneapolis, MN 55455, USA; maese004@umn.edu; 4 College of Biological Sciences, University of Minnesota, Minneapolis, MN 55455, USA; blake628@umn.edu (J.B.); sarcasmisus@gmail.com (E.Z.); nathandeep01@gmail.com (N.D.); verne036@umn.edu (M.V.); 5 Medical School, University of Minnesota, Minneapolis, MN 55455, USA; mckay228@umn.edu (J.M.); schu4336@umn.edu (S.J.S.); 6 Comparative and Molecular Biosciences Graduate Program, University of Minnesota, Minneapolis, MN 55455, USA; 7 Neuroscience Graduate Program, University of Minnesota, Minneapolis, MN 55455, USA; chan2155@umn.edu (A.C.); roman325@umn.edu (A.R.); waldr155@umn.edu (M.A.W.); kperson@umn.edu (K.P.); lil@umn.edu (L.L.); 8 Department of Experimental and Clinical Pharmacology, University of Minnesota, Minneapolis, MN 55455, USA; korth047@umn.edu; 9 Department of Veterinary Population Medicine, University of Minnesota, Minneapolis, MN 55455, USA; vdivanak@umn.edu (V.K.); naray280@umn.edu (M.N.); arju0001@umn.edu (T.A.); cheeran@umn.edu (M.C.); 10 Molecular Pharmacology and Therapeutics Graduate Program, University of Minnesota, Minneapolis, MN 55455, USA; 11 Molecular, Cellular, Developmental Biology, and Genetics Graduate Program, University of Minnesota, Minneapolis, MN 55455, USA

**Keywords:** pulmonary dysfunction, immune dysfunction, pediatric population, coronavirus, long COVID, post-acute sequelae of COVID-19, artificial intelligence, ChatGPT, large language model, SAR-CoV-2

## Abstract

Coronavirus disease 2019 (COVID-19) in adults is well characterized and associated with multisystem dysfunction. A subset of patients develop post-acute sequelae of SARS-CoV-2 infection (PASC, or long COVID), marked by persistent and fluctuating organ system abnormalities. In children, distinct clinical and pathophysiological features of COVID-19 and long COVID are increasingly recognized, though knowledge remains limited relative to adults. The exponential expansion of the COVID-19 literature has made comprehensive appraisal by individual researchers increasingly unfeasible, highlighting the need for new approaches to evidence synthesis. Large language models (LLMs) such as the Generative Pre-trained Transformer (GPT) can process vast amounts of text, offering potential utility in this domain. Earlier versions of GPT, however, have been prone to generating fabricated references or misrepresentations of primary data. To evaluate the potential of more advanced models, we systematically applied GPT-4 to summarize studies on pediatric long COVID published between January 2022 and January 2025. Articles were identified in PubMed, and full-text PDFs were retrieved from publishers. GPT-4-generated summaries were cross-checked against the results sections of the original reports to ensure accuracy before incorporation into a structured review framework. This methodology demonstrates how LLMs may augment traditional literature review by improving efficiency and coverage in rapidly evolving fields, provided that outputs are subjected to rigorous human verification.

## 1. Introduction

The COVID-19 pandemic triggered by the Severe Acute Respiratory Syndrome Coronavirus 2 (SARS-CoV-2) virus emerged as a health catastrophe of global proportions. SARS-CoV-2, a betacoronavirus, shares its genus with other related viruses like SARS-CoV and MERS-CoV. The virus possesses a positive-sense RNA genome and utilizes ACE2 and TMPRSS2 for host cell entry. Since the first reported case of SARS-CoV-2 in Wuhan, China in late 2019, over 767 million people have been infected globally, resulting in nearly 7 million deaths [1]. Moreover, it has been estimated that among those who survive the infection, at least 65 million individuals worldwide have persistent debilitating effects of long COVID [2]. 

Although SARS-CoV-2 infection is usually mild in children, some develop persistent or delayed complications. Post-Acute Sequelae of SARS-CoV-2 (PASC, or long COVID) is defined as symptoms lasting beyond 4–12 weeks post-infection that affect daily functioning [3], while Multisystem Inflammatory Syndrome in Children (MIS-C) is a rare, hyperinflammatory condition occurring 2–6 weeks after infection, often involving cardiac, gastrointestinal, and mucocutaneous systems [4]. Early recognition and management of both conditions are critical to reduce morbidity and guide long-term monitoring.

The consequences of long COVID have been thoroughly documented in the adult population where multiple organ systems such as the lung, immune, brain, and heart can be severely affected. Long COVID can affect multiple organ systems and lead to significant impairment of function due to organ damage [5]. Studies suggest that 17–45% of infected adults still experience persistent symptoms 3–4 months after infection [6,7]. Approximately 8.4% of U.S. adults have reported ever experiencing long COVID. At the time of the survey, 3.6% of adults indicated that they currently have long COVID, and 2.3% reported that their symptoms limited their daily activities [8].

A recent study by Peter et al. followed patients initially identified with long COVID 6–12 months after SARS-CoV2 infection, and then for an additional 12 months [9]. The authors observed that 68% of patients with long COVID during the initial period still exhibited symptoms of long COVID in the following year.

New reports are chronicling the global impact of COVID and long COVID in the pediatric population including the organ systems affected by SARS-CoV-2 infection [10,11,12,13]. Long COVID in children represents a broad spectrum with MIS-C representing the severe form [14]. However, MIS-C is a rare complication with an incidence of only 3.27 per 100,000 children [15]. Efforts to identify PASC in children are necessary to facilitate appropriate management and intervention strategies [16].

**Figure 1:**
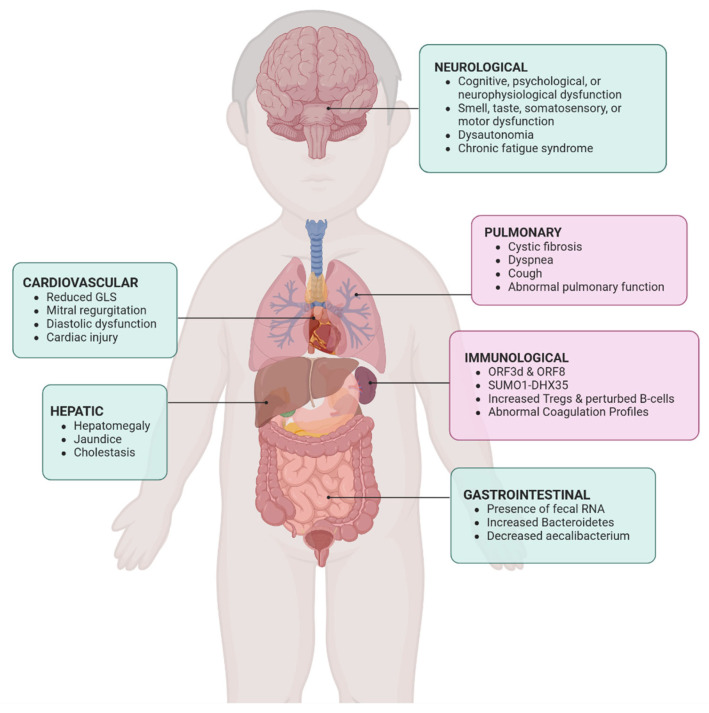
Pulmonary and immunological complications of long COVID. DHX35 = DEAH-Box Helicase 35; GLS = Global Longitudinal Strain; ORF3d = Open Reading Frame 3d (SARS-CoV-2 gene); ORF8 = Open Reading Frame 8 (SARS-CoV-2 gene); SUMO1 = Small Ubiquitin-like Modifier 1; Tregs = Regulatory T cells.

Like in the adult population, SARS-CoV-2 is transmitted through the air from person to person, leading to the initial major symptoms affecting the lung and pulmonary function. Infected patients can exhibit persistent coughing, and later difficulty in breathing, resulting in low oxygen saturation levels in the blood. In addition to pulmonary dysfunction, initial reports found immunological, neurological, cardiovascular, gastrointestinal, and hepatic complications in pediatric populations (Figure 1). Long COVID syndrome involves lingering mild-to-moderate symptoms and several medical complications. A comprehensive summary of the current understanding of long COVID has been provided, describing more than 50 long-term effects of COVID infection, including but not limited to fatigue, cognitive impairment, altered perception of smell and taste, autonomic dysregulation, thermoregulation abnormalities, and gastrointestinal disorders [10,17].

The number of reports focusing on COVID and long COVID has grown tremendously since the first reports of the SARS-CoV2 outbreak in China, and it has now become nearly impossible for one individual to read and digest every report on this topic. In order to stay abreast of key research developments in science, new technologies such as artificial intelligence (AI) and large language models (LLMs) have been proposed to generate accurate and factual reviews and reports. LLMs are advanced AI systems designed to understand, generate, interpret, and respond to human language in a manner that mimics human-like understanding. LLMs are a type of machine learning model within the broader category of natural language processing (NLP). LLMs are large in terms of the size of the neural networks that they are based on and the massive amounts of data they are trained on, including datasets of texts from the internet, articles, books, and other written materials. LLMs such as GPT (Generative Pre-trained Transformer) can perform a wide range of tasks such as text generation, translation, and summarization. At the time of this writing, ChatGPT-4 was trained on biomedical articles listed in Public Medline (PubMed) up to the year 2021.

LLMs have been increasingly used in the field of medicine offering innovative approaches to improve medical education, patient care, and research. In the future, they may provide clinical decision support by understanding and interpreting complex medical data [18]. LLMs may support medical education by providing students with access to a vast knowledge base, interactive learning experiences, and simulation-based training scenarios, and as a training assessment tool [19]. They also may serve as virtual assistants offering 24/7 support to patients, answering their queries and providing guidance to health issues [20] and facilitate research in gene-gene interactions and gene regulatory networks [21].

Despite the promise of LLMs, concerns have been raised regarding the generation of misinformation. Some primary concerns include inaccuracies in generated content, opacity of training data, and model bias [22]. In addition, “hallucinations” of false information and references have been observed in LLMs [23]. To mitigate these concerns, strategies need to be explored to include fact-checking mechanisms. To address this goal, we have constrained ChatGPT-4 to analyze the results of scientific articles related to pediatric COVID that are derived from PubMed, a database of scientific reports listed by the National Institutes of Health consisting of articles published in certified journals. The authors have pre-selected articles for summary analysis by ChatGPT-4 to ensure an accurate and factual comprehensive review of the multifaceted dimensions of COVID and long COVID in children and adolescents.

Figure 1 illustrates a system-level look at complications across the body reported in pediatric patients afflicted with long COVID, including cardiovascular, hepatic, neurological, pulmonary, immunological, and gastrointestinal systems. In-depth analysis of pulmonary and immunological complications is provided below. 

## 2. Methods

The scientific literature on COVID was interrogated using PubMed, a database maintained by the National Institutes of Health for the retrieval of biomedical and life sciences literature using the keyword ‘COVID.’ We found 27 articles published in 2019; 91,677 articles in 2020; 139,011 articles in 2021; 128,736 articles in 2022; 86,957 articles in 2023, and 58,032 articles in 2024. We focused our analysis on publications in 2022 through January 2025 and refined our search strategy to long COVID in children and adolescents. This provided a core of publications that were then sub-grouped into pulmonary and immunological consequences associated with COVID. The publications in each sub-group were assigned to individuals in writing (Team A). These individuals reviewed each publication to determine whether each article was an appropriate report for inclusion in our review: (1) confirming that the references cited are authentic reports that can be found in PubMed, and (2) the information related to each reference conforms with the information presented in the original articles. Portable Document Files (PDFs) of articles included in our final list were converted into Microsoft Word format and the results section of each study was fed into ChatGPT-4 to summarize the data. We initially used ChatGPT-3 for the analysis of the articles, but found ChatGPT-4 to be superior in its depth of analysis, better comprehension of technical scientific language, improved summarization accuracy, and cross-referencing of findings across multiple studies. The summary of each article was reviewed by a reader on Team A to determine whether ChatGPT-4’s analysis was an accurate representation of the data presented in the article. If so, this information was uploaded into a master copy of the working document for our review under the appropriate subheading. The assembled document was reviewed by members of Team B where each member was assigned to review specific sections to determine whether each cited reference was listed in PubMed, and whether the ChatGPT-4 summary of the article results accurately reflected the data from the original article. This process ensured that ChatGPT-4 would not be able to include articles not selected by Team A, or fake articles, and verified that the summaries generated by ChatGPT-4 accurately represented the results of each study.

We also manually included articles that we found were necessary to produce a high-quality manuscript for publication. To account for bias, Team A and Team B were blinded to the results of the findings as well as which articles were manually sourced and summarized. All activities of ChatGPT-4, Team A and Team B were dual-reviewed by the lead authors for accuracy, consistency, and removal of plagiarism, by extensive manual revision of sections, as one would do when following best practices for drafting a scientific manuscript.

### Rubric for Evaluating ChatGPT-4-Provided Articles

We developed a 5-point scoring rubric to evaluate the accuracy and relevance of AI-generated research article summaries, adapting criteria from [24] for the authors to score. The rubric assessed whether the articles provided by ChatGPT-4 accurately reflected the source material, directly addressed the specific research topic, were actively listed in PubMed, and existed on the publisher’s website. Each criterion was scored on a 5-point scale where 0 = the criterion was not met at all and 5 = the criterion was fully met. Table 1 provides the criteria and scoring descriptors.

A summary of the results of the rubric scoring is provided in Table 2. Across the evaluated articles, the mean accuracy score for ChatGPT-4 summaries was 4.2 ± 0.9, with 82% achieving a score of 4 (Good) or greater. Publisher website availability scored highest (mean 4.9 ± 0.4), PubMed listing scored second highest (4.9 ± 0.8), while relevance to topic scored second lowest (4.6 ± 0.7) above the accuracy of summaries. In all, the percent scoring 4 or greater was between 81 and 98% for all criteria, and the percent scoring 5 was between 51–98%.

## 3. Pulmonary Complications

As with the adult population, the initial impact of SARS-CoV-2 infection results in long-term complications in pulmonary function in children. Ipek et al. evaluated pulmonary function in children with COVID-19 in a cohort consisting of 34 children and a control group of 33 children [25]. Results showed that the forced vital capacity (FVC), a measurement used in pulmonary function tests that represents the total amount of air a person can forcefully exhale after inhaling as deeply as possible, and the forced expiratory volume (FEV), another pulmonary functional test that represents the amount of air a person can forcefully exhale during the first seconds of a forced breath, values were significantly lower in the patient group compared to the control group. These findings demonstrated that children and adolescents who had recovered from SARS-CoV-2 infection continue to exhibit impaired pulmonary function.

### 3.1. Persistent Pulmonary Symptoms

Öztürk et al. examined respiratory function of pediatric patients 3 months after COVID-19 infection [26]. Of the 50 patients assessed, the major demographics had a median age of 15 years and were predominantly male. Respiratory symptoms were present in 14 (28%) patients, with dyspnea and exertional dyspnea being the most common. Four patients who did not have respiratory symptoms at active infection developed these symptoms at follow-up, 3 patients had an obstructive deficit, 1 had a restrictive deficit, and 4 patients had impaired diffusion capacity (DLCO). The only significant difference in patients with respiratory symptoms was a lower lymphocyte count. PFTs showed significantly lower FEV1/FVC and higher lung clearance index (LCI) in patients with respiratory symptoms. In patients with severe disease, 50% reported persistent respiratory symptoms compared to 12.5% in patients with non-severe disease. The DLCO was significantly lower in the severe disease group, but LCI was similar between the two groups. While no risk factors were detected in multivariable analysis, univariable analysis showed that lymphocyte count and elevated d-dimer were associated with persistent respiratory symptoms.

The study by Al-Shamrani et al. in 2023 focused on pediatric patients in Saudi Arabia who had previous COVID infections [27]. The study included 194 patients, with the majority being Saudi citizens from its southern region. The age distribution of patients was as follows: 6–14 years (51.0%), 3–5 years (32.3%), and younger than 2 years (16.7%), with females accounting for 41.7%. Nearly half of the patients (48.5%) had a previous SARS-CoV2 infection in 2022, and a small percentage (2.1%) were infected in 2019. Among the patients, 13.9% required hospital admission, and 4.2% needed intensive care treatment. After 4 weeks post-infection, 92.2% of patients still reported persistent symptoms. The most common symptom was cough, reported by 69.8% of patients, with 12.3% experiencing both cough and wheezing. The majority of patients described the cough as dry and nocturnal.

Residual cough affected school attendance and daily activities for 39.3% of patients, with 31.1% reporting associated chest pain, 51.9% associating it with wheezing, and 27.1% associating it with shortness of breath. Most patients (54.4%) reported a residual cough lasting less than one month, while 31.4% reported a duration of 1–2 months, and only 1.0% experienced a cough lasting more than 3 months. Residual cough following COVID was commonly observed, lasting at least 2 months, and shared characteristics with coughs typically seen in asthma patients. This lingering cough negatively impacted daily activities and school attendance, with a notable number of siblings in the same families also being affected.

Bode et al. investigated the impact of SARS-CoV-2 infection on spirometry parameters and persistent symptoms [28]. More than half of the participants in the study had positive SARS-CoV-2 serology at 4 months post infection. The results showed that adults were seropositive and symptomatic significantly more often during acute infection than adolescents and children were. However, there were no statistically significant differences in spirometry parameters between SARS-CoV-2 seropositive and seronegative participants in the whole cohort or in the subgroups of children, adolescents, and adults. Questionnaires regarding persistent symptoms after SARS-CoV-2 infection 12 months after initial SARS-CoV-2 infection showed that adults reported more persistent symptoms compared to adolescents and children.

A study characterizing long COVID in children and adolescents found that long COVID manifests differently in adolescents compared to younger children [29]. The study analyzed data from over 5000 participants across more than 60 sites nationwide. Findings indicate that adolescents (ages 12–17) predominantly experience symptoms such as low energy or tiredness, while school-age children (ages 6–11) are more likely to report headaches. These distinctions suggest that a uniform approach may not be effective in identifying and treating long COVID in pediatric populations, underscoring the need for age-specific diagnostic and therapeutic strategies.

### 3.2. Pulmonary Comorbidities and Risk Factors

Pulmonary Comorbidities and Obesity. An overview of the progression of SARS-CoV-2 infection in pediatric populations from 2021 to 2022 was explored [30]. Demographics included 101 patients who tested positive for SARS-CoV-2, of which 67 had presentations consistent with acute symptomatic infection. The majority of patients had an extrapulmonary comorbidity, and a history of pulmonary comorbidity and obesity was significantly associated with markers for severe disease. The study also found that a history of underlying lung disease and acute severe COVID was associated with long-term chronic complications. Respiratory illnesses like pneumonia can be caused by COVID and can lead to respiratory symptomatology [31].

The association of body mass index (BMI) with increased risk of developing long COVID among children and young adults was explored in a cohort of 172,136 participants across 26 U.S. children’s hospitals between March 2020 and May 2023 [32]. Findings revealed that elevated BMI was significantly associated with an increased risk of long COVID in a dose-dependent manner. Specifically, compared to peers with a healthy BMI, those with obesity had a 25.4% higher risk, and those with severe obesity had a 42.1% higher risk of developing long COVID. These results suggest that obesity is an important risk factor for pediatric long COVID, highlighting the need for targeted care to prevent chronic conditions in at-risk children and young adults who have been infected with SARS-CoV-2.

Pneumonia. A study assessed the development of pneumonia in pediatric COVID [33]. This study included 22 patients from 17 families with inherited MyD88 or IRAK-4 deficiency. The patients were from different countries, including Spain, USA, Belgium, Germany, Canada, Morocco, Italy, and Switzerland. All 22 patients were infected with SARS-CoV-2 before vaccination—confirmed through real-time PCR or antigenic assays. The patients ranged in age from 2 months to 24 years, with a mean age of 10.9 years. Among the patients, 16 were receiving prophylaxis at the time of SARS-CoV-2 infection, including oral antibiotics and IgG replacement therapy. Sixteen of the patients were hospitalized for pneumonia, with varying severity. Six patients had moderate pneumonia, and 10 had hypoxemic pneumonia, requiring admission to an intensive care unit (ICU) in 6 cases. Patients infected with other viral variants had a wide spectrum of clinical manifestations, ranging from silent infection to death; however, none of the patients infected with the Omicron variant suffered from pneumonia.

The risk of severe infection, hospitalization, ICU admission, and mortality was significantly higher in MyD88- or IRAK-4-deficient patients compared to the general population. Despite their genetic deficiencies, the MyD88- or IRAK-4–deficient patients were able to mount an inflammatory response to SARS-CoV-2 infection, including the production of type I interferons (IFNs) by other cells. Neutropenia was frequently observed in MyD88- or IRAK-4-deficient patients during acute SARS-CoV-2 infection. The possibility of codominance for the COVID-19 phenotype was explored in heterozygous carriers of MYD88 or IRAK4, but no significant differences in disease severity were observed in these individuals.

Cystic Fibrosis. Boguslawski et al. examined comorbidities with lung diseases, including 41 children with COVID [34]. Seven of the children (17.1%) reported persistent symptoms, with decreased exercise tolerance being the most common (57.1%), followed by dyspnea and cough (both reported by 42.9%). Lung ultrasound examinations revealed coalescent B-lines, caused by discrete vertical reverberation artifacts in the pleural line, in 37% of the children and small subpleural consolidations in 29%. The extent of lung ultrasound (LUS) abnormalities was significantly greater at the first follow-up visit than at the second. There were no significant differences in the PFT results between the study group and healthy children, nor were there any significant differences in PFT results between the two follow-up visits in the study group. Children with cystic fibrosis have been found to be impacted by SARS-CoV-2 similarly to the general population, with most children reported to be asymptomatic. Radiological assessments in children have shown a high incidence of abnormalities, including ground-glass opacities, bilateral lung lesions, pulmonary nodules, patchy shadows, halo signs, consolidation, air bronchogram signs, cord-like shadows, crazy-paving patterns, and pleural effusion.

These results suggest that patients with pre-existing lung disease are at a higher risk of developing long-term complications following SARS-CoV-2 infection. Microvascular damage and thrombosis are known complications of severe COVID, involving elevated levels of neutrophils in the lungs of patients with severe COVID [35]. These studies emphasize the need for ongoing monitoring of patients with a history of severe COVID, particularly those with underlying lung disease, to identify and manage any long-term chronic complications.

### 3.3. Long-Term Pulmonary Sequelae

Long-term sequelae of COVID were examined by Palacios et al. in evaluating 82 adolescents at a median of 3.5 months after SARS-CoV2 infection [36]. About 80% of the patients reported two or more symptoms at clinic presentation, which included cough, chest pain, dyspnea at rest, and exertional dyspnea. At follow-up, exertional dyspnea persisted for most patients. Clinical phenotypes identified in the study included inhaled corticosteroid responsiveness, paradoxical vocal fold motion disorder, deconditioning, and dysautonomia. Obesity, anxiety, and resting dyspnea were associated with reduced performance capacity, while female sex and resting dyspnea were associated with difficulty in breathing and fatigue.

The study by Campos et al. reviewed 17 articles reporting on the lasting effects of COVID-19 on children and adolescents [37]. These studies were conducted in various countries, with a total of 124,568 post-COVID-19 children and adolescents participating between 3 and 12 months after symptom onset or hospital discharge. The study participants’ age range was between 0 and 19 years, with 48% being male. The prevalence of long COVID in children and adolescents based on 12 studies was 30%. Risk factors contributing to long COVID in this population were female sex, older age, multiple symptoms during acute infection, longer hospital stays, and respiratory distress following acute COVID-19.

Lung imaging and pulmonary function assessments revealed notable findings among the participants. Chest CT scans and X-rays identified abnormalities in 10% of individuals, while pulmonary function tests (PFTs) showed irregularities in 24%, including 7% with an obstructive pattern and 5% with impaired diffusion capacity. Regarding cardiorespiratory symptoms and fatigue, 6% of participants reported experiencing chest pain or tightness, and another 6% noted heart rhythm disturbances or palpitations. Breathing difficulties and dyspnea affected 16%, while sore throat was reported by 10%, persistent cough by 4%, and rhinorrhea by 2%. Prolonged fatigue emerged as a significant concern, impacting 24% of participants. In terms of exercise capacity and daily functionality, 20% reported reduced exercise tolerance, and nearly half (48%) experienced functional limitations, including challenges with performing routine daily activities. These findings suggest that, much like in adults, children and adolescents with long COVID may experience lingering abnormalities in lung imaging and function, cardiorespiratory symptoms, fatigue, reduced exercise capacity, and diminished functional abilities within 3 to 12 months following infection.

### 3.4. Summary of Pulmonary Consequences

**Figure 2:**
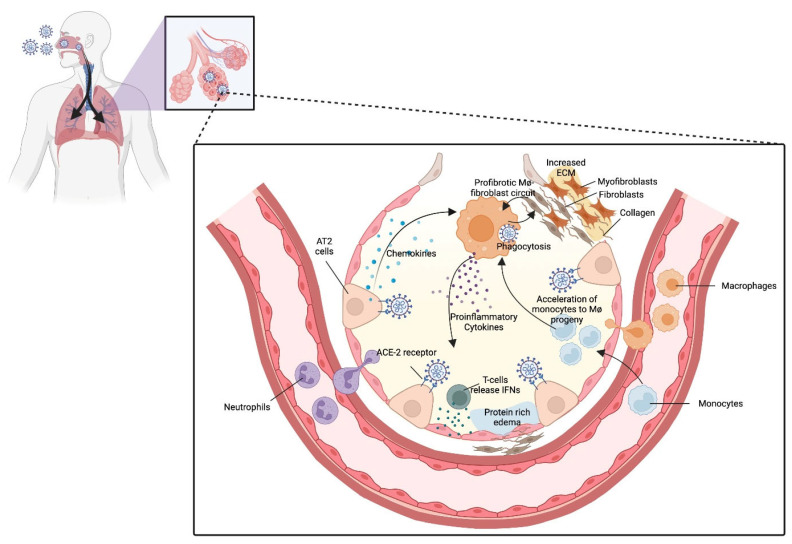
Cellular mechanisms of pulmonary dysfunction in long COVID. SARS-CoV-2 infection affects lung alveoli and leads to inflammation and fibrosis. While immune cells attack the virus, this process drives inflammation, fluid buildup, and excessive tissue remodeling, which can progress to lung fibrosis and long-term damage. ACE-2 = Angiotensin-Converting Enzyme 2 (receptor used by SARS-CoV-2 to enter cells); AT2 = Alveolar Type II (epithelial cells in the lung alveoli); ECM = Extracellular Matrix; IFNs = Interferons; MΦ = Macrophages.

COVID is a complex disease with a wide range of clinical outcomes, from no symptoms to severe respiratory failure and death. The progression and treatment of COVID can be better understood by considering the location of the infection and the types of cells affected throughout the disease course. Studies also found that the lethality of SARS-CoV-2 is correlated with the severity of pulmonary pathology, observed in both animal models and in humans [38]. Initially, the virus infects and replicates in the sinonasal airway epithelium (Figure 2), which consists of ciliated and mucus-secreting cells responsible for clearing particles from the airways primarily targeting cells for early replication and release [39,40]. The likelihood of death and long-term complications from SARS-CoV-2 infection largely depends on the damage to the alveolar epithelium. One notable factor in the prolonged effects of COVID is the harm to progenitor cells responsible for regenerating the epithelial cells in both the conducting airways and alveoli. In the conducting airways, basal stem cells are typically unaffected, while in the alveoli, type II cells often sustain damage, while in some patients, the repair of the alveolar barrier leads to excessive fibrosis [41]. Severe COVID patients in adults develop pneumonia and hyperinflammation as a result of a macrophage activation syndrome comprising a mixture of cytokines that lead to downstream fibrotic remodeling in the lung [42]. Potential biomarkers of post-COVID pulmonary fibrosis include elevated serum levels of specific extracellular matrix molecules when compared between healthy individuals and patients with sudden worsening of pulmonary fibrosis [43].

SARS-CoV-2 affects the lung, eliciting aggressive inflammatory responses that can eventually lead to respiratory failure in severe cases. COVID has been linked to pulmonary fibrosis due to the abnormal healing of the injured lung parenchyma [44,45,46,47], which can result in lung function impairments characterized by a reduction in carbon monoxide diffusion capacity 6 months after disease onset [48]. This may be caused by improper inflammatory response and thromboembolic events that result in endothelial dysfunction.

Monocytes and macrophages are major sources of inflammatory cytokines and interact with lung-resident T-cells, lead to impairment of alveolar regeneration, and correlate with severe COVID symptoms [49,50,51]. A study in a humanized mouse model demonstrated a negative correlation between splenic T cells and transcriptional changes in lungs, which suggest a stress response in the lungs that may impact systemic lymphopenia [52]. Another study suggested pulmonary redox imbalance occurring in the early stages of COVID-19 acute respiratory syndrome may drive fibroproliferation, hindering the recovery from lung function impairment and leading to the increase in the risk of death [53]. Most studies however highlight the necessity for further research on the impact of long COVID on children and whether pulmonary specific examinations can be predictive of long COVID [54].

In long COVID, resident alveolar macrophages (AMs), defined by the expression of FABP4 and MARCO, are shown to be significantly lowered. Since resident AMs are able to move through the alveoli, it is thought that they are responsible for the spread of SARS-CoV2 within the lung. Theories posited in the emerging literature have varied from direct infection of SARS-CoV2, a hyper-inflammatory environment, or a loss of survival signals cued by the death of epithelial cell damage/death. When infected, resident AMs have been found to have high levels of IL-1ꞵ, CCL4, CCL20, CXCL10 and CXCL11 that lead to the recruitment of monocytes and T-cells in the bronchoalveolar space. Current evidence also points to macrophage recruitment from these derived monocytes, but it is still unclear if this differs between those expressing pro-fibrotic features (SPP1, TREM2) and pro-inflammatory features (CCL2, CCL3, CXCL10). Elevated chemokine production creates a positive feedback loop to recruit more monocytes, thereby maintaining the inflammatory response. The significance of macrophages expressing pro-fibrotic features has yet to be shown, but evidence points to an attempted response to repair the damaged epithelium [55].

## 4. Immunology of Pediatric COVID-19

Several studies propose a pathogenesis model for long COVID driven by persistent viral infection or a component of the virus, leading to striking pathologic systemic inflammation [56,57]. There are three phases of the immune response to SARS-CoV-2 infection, including the initial innate immune response, the adaptive immune encounter with T and B cells, and the cytokine storm associated with excessive release of pro-inflammatory cytokines and tissue injury [58]. Infection also has been shown to induce the development of autoantibodies and increased high-affinity anti-spike antibodies, which persist in some patients and contribute to long-lasting symptoms [59,60,61,62].

The initial innate and adaptive immune response in long COVID is similar to those seen in the primary encounter of a host with an acute microbial pathogen but with the continued presence of the microbial antigen resulting from the persistent infection. This can continue to stimulate both the innate and adaptive immune systems, leading to the development of long COVID. The consequences of the intense inflammation and immune dysregulation associated with persistence of SARS-CoV-2 or its structural proteins on disease manifestations for patients with long COVID are related to diffuse endothelial damage and microthrombosis, which consequently lead to organ damage [63,64].

Antibodies also have been implicated in the development and persistence of long COVID, suggesting a possible role for the immune system’s response in contributing to the prolonged symptoms observed in some individuals following SARS-CoV-2 infection. In one such study, Hachim et al. examined the antibody responses to SARS-CoV-2 in infected children and adults [65]. Using the Luciferase Immuno-Precipitation System, the investigators found that the antibody specificity of COVID in children is distinct from infected adults, with children having lower levels of S1 and S2 antibodies and higher levels of N and E antibodies. They also identified a combination of ORF3d and ORF8 antibodies that most accurately distinguishes infected samples from uninfected controls, and investigated the relationship between antibody production and cytokine profile.

Children also were found to have a slower rate of decay in their IgG antibody response in a study by [66,67] also found children with a similar ability as adults in making and sustaining neutralizing antibodies; however, ref. [66] found that children were able to maintain higher neutralizing antibody levels long-term. Another study by [68] found that children were able to maintain relatively stable neutralizing antibody titers over time, but there was largely no neutralization of the Omicron variant. When vaccinated, the concentrations of antibodies in children with long COVID differ from those who are unvaccinated. A study conducted by [69] identified key factors associated with long-term symptoms following SARS-CoV-2 Delta variant infection in children and adolescents, further supporting the observation that participants had spike-specific IgG antibodies and were seropositive for HAT antibodies at 3 months post-infection [66,67,68,69]. Vaccinated individuals had higher convalescent antibodies compared to unvaccinated subjects, but no difference in antibody titers in respect to persisting symptoms. Older age, the presence of acute symptoms, and higher antibody titers were significantly linked to persistent symptoms, extending findings previously reported in adults to younger populations. This suggests that immune dysfunction may play a role in maintaining symptoms in children, similar to observations in adolescents after other viral infections.

The functions of T cells, critical components of the adaptive immune system, are significantly disrupted by COVID, which may contribute to the immune dysfunction observed in acute and long-term SARS-CoV-2 infection. Children have been found to have a different T cell profile compared to adults [67]. Specifically, ref. [68] observed that antigen-specific CD4 T cells remained stable over time while CD8 T cells declined in children after recovery from COVID. However, children possessed perturbed distributions of B-cell subsets and expanded regulatory T cells at the onset of COVID, and the persistence of COVID symptoms correlated with a reduction in B cells and an unstable balance of regulatory T cells as well as in long COVID [70].

Some studies have delved into the ways in which COVID alters the immune system, with particular attention given to its effects on hematopoietic stem and progenitor cell (HSPC) populations. There is significant expansion of the hematopoietic stem cell and multipotent progenitor (HSC/MPP) subset following severe disease during early convalescence of 2 to 4 months, with the long COVID cohort demonstrating a more pronounced version of this phenomenon, suggesting the emergence of an emergency hematopoiesis phenotype [71]. Using flow cytometry to confirm this finding, the frequency of long-term hematopoietic stem cell subsets was notably elevated. This study also found an increased frequency of neutrophil progenitors in those with long COVID, suggesting alterations in HSPC behavior, likely due to specific genes within the HSC/MPP subcluster. Gene ontology analysis revealed genes linked to granulopoiesis, inflammatory response, and neutrophil migration programs, offering insights into the mechanisms underpinning the observed shifts in neutrophil production.

An additional alteration in the immune system following COVID involves the dysregulated regulation and activity of AP-1 transcription factors (TFs) and interferon regulatory factors (IRFs). Both AP-1 and IRFs are critical players in the innate and adaptive immune responses, orchestrating the regulation of genes essential for immune activation, antiviral defense, and inflammation. The disruption of their activity during and after SARS-CoV-2 infection reveals key insights into how the virus perturbs immune signaling pathways, contributing to immune dysfunction and potentially to long COVID symptoms [71]. Both HSPCs and monocytes had diminished expression, likely due to a potent negative feedback program for inflammation. However, IRF activity remained persistent, suggesting a shift from AP-1-associated inflammation to IRF-driven antiviral activity over time [72]. These studies show that children have different immune responses to SARS-CoV-2 compared to adults, and that the generation and persistence of immune responses may be age-related.

### 4.1. Immunological Mechanisms

Studies interrogating the alterations in immune cell responses are providing insights into the underlying mechanisms of long COVID. Various changes in antibody concentrations have been found across multiple studies. Cytokines that were overexpressed expressed included type I (IFN-β) and type III (IFN-λ1) cytokines, along with other pro-inflammatory cytokines [73,74]. Lymphocyte concentrations were found to be increased in those with long COVID, which may indicate that the immune system is still activated and the virus may still persist within the body [73,74,75]. In addition to cytokines and lymphocytes, concentrations of interferons, monocytes, mast cells, eosinophils, and some pro-inflammatory mediators were found to be higher, while the concentration of interferon-γ-induced protein 10 was decreased [73,74,75]. These altered concentrations suggest that immune dysregulation is associated with long COVID. This is further supported by the over activity of the immune system during long COVID, leading to the production of autoantibodies such as anti-nuclear, anti-neutrophil cytoplasmic, and anti-interferon antibodies [73,74]. While it is currently unknown why this autoimmune response exists with long COVID, the study by [73] suggests that it may be due to neutrophil extracellular traps.

The response to SARS-CoV-2 infection also can result in the production of various auto-antibodies, including anti-apolipoprotein A-1 IgGs (AAA1), which may contribute to long-term symptoms. One study followed 200 hospital employees who had been infected with SARS-CoV-2 for up to 12 months to investigate the persistence of symptoms and the evolution of AAA1 IgG levels over time [76]. The study found that higher initial AAA1 IgG levels were associated with a faster decrease over time and that older age, comorbidities, and higher body mass index (BMI) were associated with higher initial AAA1 IgG levels. AAA1 IgG levels at 1-month post-infection predicted persistent respiratory symptoms at 12 months, and positive AAA1 IgGs at 6 and 12 months post-infection were associated with the persistence of symptoms of any kind and respiratory symptoms at 12 months. While AAA1 IgGs induced a significant increase in IFN-α production, it did not have a significant effect on IFN-γ production.

Autoantibodies in patients with long COVID were studied by [77]. By analyzing plasma samples from patients with long COVID, they found that autoantibodies against SUMO1ylated isoform DEAD/H box helicase 35 (SUMO1-DHX35) were present in 9.8% of patients with long COVID, with no evidence of them in healthy children or children tested after SARS-CoV-2 infection. They also found that these autoantibodies were exclusively bound to SUMOylated DHX35 but not non-SUMOylated DHX35, and concluded that autoantibodies against SUMO1-DHX35 may be associated with long COVID. 

Studies have shown that the antibody response to long COVID appears to differ between children and adults, and that the time to viral clearance and changes in viral load over time differed between the two groups [78]. In addition, ref. [78] showed the median time to viral clearance was shorter in children than in adults, and that viral load decreased over time since infection and remained below the threshold considered necessary for transmission.

Researchers also studied the activation of immune response pathways, particularly the RLR-mediated innate response and interferon (IFN) signaling. Earlier variants, such as B.6 and B.1.1.8, activated these pathways, leading to the induction of interferons and interferon-stimulated genes (ISGs) [79]. However, the Delta and Alpha variants of SARS-CoV2 evaded the IFN response and had varying levels of susceptibility to RLR activation. Poly(I·C), a synthetic analog of viral RNA, partially activated RLR pathways and restricted viral replication. Furthermore, the study observed that Delta infection caused a more intense and persistent subversion of cytokines, chemokines, and antigen presentation genes compared to the Alpha variant, suggesting a potential mechanism for the Delta variant’s ability to evade host immune responses. Overall, the research revealed distinct differences in the replicative and infectious fitness of different SARS-CoV-2 variants and highlighted the complex interactions between viral replication and host immune responses, shedding light on the factors that contribute to the spread and impact of different variants of the virus.

### 4.2. Summary of Immunological Complications

**Figure 3:**
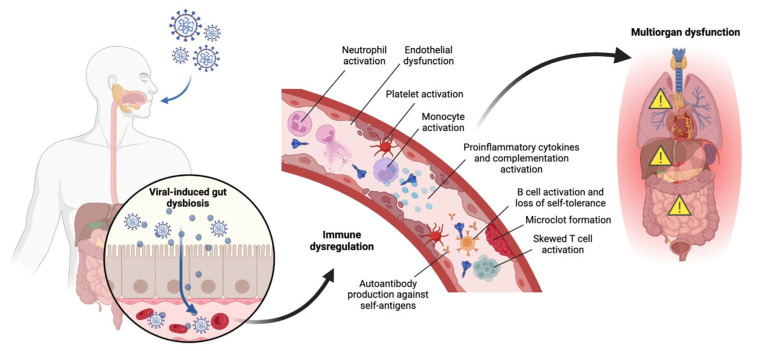
Immunological mechanisms in long COVID with SARS-CoV-2 reservoir in the gut. Immune system dysfunction consists of antibody production of self-antigens, complement activation, release of pro-inflammatory cytokines, activation of B cells, neutrophils, platelets, monocytes and skewed T cells. Alterations in the immune system can lead to multi-organ dysfunction.

Recent studies suggest that following the infection of the lung by SARS-CoV-2, the virus can infect the gut [80,81] and potentially establish a reservoir for viral reproduction and immune cell dysfunction (Figure 3). Alterations in immune cell dysfunction, in turn, can lead to multi-organ dysfunction. In advanced stages, innate immune activation, inflammatory myeloid cells, and TGFβ signaling affect fibrosis progression and impaired lung repair [82,83], leading to chronic pulmonary epithelial and immune cell dysfunction [84]. Patients with persistent pulmonary sequelae had heightened activation of neutrophils in circulation for up to 6 months [85,86]. Animal models demonstrated an increase in SPP1-expressing macrophages, associated with lung fibrosis, while blocking SPP1 reduced mortality and inflammation, indicating its potential as a therapeutic target [83]. These findings suggest that targeting TGFβ and SPP1 pathways could prevent excessive systemic inflammation and fibrosis in severe COVID cases. Certain proteins like YKL-40 and MR-pro-ADM, which are engaged in inflammation and lung fibrosis, have been correlated with neurological complications after COVID infection [87]. Long COVID is also associated with epigenetic changes, and regulation of miRNAs and lncRNAs downregulate pro-inflammatory cytokines and relieve acute symptoms [88]. Even though advanced age is the main risk factor for poor outcomes associated with bronchial dilations and the recovery of lung parenchyma [89,90], treatments that target the early stages of pulmonary fibrosis can be explored further. SARS-CoV-2 has also been shown to induce transcriptome and proteome profiles to macrophages associated with idiopathic pulmonary fibrosis, with viral transcripts detected in pulmonary macrophages. These findings suggest that the profibrotic macrophage phenotype may be a direct consequence of SARS-CoV-2 infection in severe COVID-19 [91].

The findings collectively emphasize that COVID-19, particularly in its severe forms, induces persistent immune and inflammatory changes that contribute to the development of lung fibrosis and other long-term complications. Targeting key pathways such as TGFβ and SPP1, along with exploring epigenetic interventions, holds promise for mitigating these long-term effects. Additionally, focusing on the early stages of fibrosis progression may offer therapeutic opportunities to prevent or alleviate the chronic pulmonary dysfunction and other multisystemic complications seen in post-COVID patients. Understanding the molecular and cellular changes induced by SARS-CoV-2 will be critical for the development of effective treatments and the management of long COVID.

## 5. ChatGPT-4 Implementation and Guidelines for Generating Scientific Reviews

The method of selecting journal articles for the review consists of manual involvement to avoid the generation of fake studies. The journal articles included were selected by the authors after reading the abstracts to assess the appropriateness of each article. We found that Chat GPT-4 performed well in summarizing the results of scientific studies related to long COVID in the pediatric population. LLMs such as Chat GPT-4 can be constrained from providing false reports by manually supplying the results sections from articles published in the scientific literature. To automate this process in the future, LLMs should be constrained to using databases like PubMed where published reports have been peer-reviewed and provided by authentic journals. Moreover, it is critical that authors using LLMs for assisting with publishing scientific reviews verify that the articles cited are real and not fabricated, and ensure that the summary information provided for each article is an accurate representation of the original articles. This should be part of the responsible conduct of authors who employ LLMs in writing scientific reviews and of journal editors accepting these types of scientific reviews for publication.

Currently, there exists LLM-based software that can assist with literature review such as Consensus, Elicit, SciSpace, Semantic Scholar, and Research Rabbit and general-purpose search like Perplexity (Supplementary Table S1). These tools help to streamline the process of identifying, summarizing, and citing research articles across various academic domains using natural language queries. Consensus locates research articles and automatically generates an overall and individual summary for the retrieved articles with links to each. Another option is Elicit, a LLM application that generates articles related to a query, generating a summary of “top” citations—by some metric of relevance and/or accuracy—with the option to further filter results. It displays the retrieved articles in tabular format alongside an AI-generated summary of each article’s abstract or other section like “limitations” or “algorithms,” facilitating cross-checking. Elicit, along with others like Semantic Scholar, include a beta chatbot that allows discourse about selected papers (e.g., “What are the limitations of these papers?”). For more a general-purpose search, Perplexity is an AI research assistant built on a chosen state-of-the-art generative model by providers like OpenAI (OpenAI, 2023) and Anthropic [92]. Adjusting the search accordingly, Perplexity retrieves information from only academic sources. Furthermore, applications like OpenAI’s chatbot ChatGPT-4o and other specialized applications, including Enago Read, allow the user to manually upload references and retrieve AI-generated summaries, including AI assistants, chatbots or co-pilots that facilitate efficient information retrieval through additional prompts like instructions, question-answering, etc. Similarly to ChatGPT-4, performance improves with clear and specific prompts, providing sufficient context to assist the LLMs under the hood in generating their response.

### Future Direction and Ensuring Research Integrity

As GPTs can generate a response regardless of lack of confidence or inaccuracy, these techniques can help reduce hallucinations by using a sufficiently powerful model (e.g., higher number of parameters and tokens trained on, greater context window, etc.), post-training via SFT and RL (e.g., more training on examples in domain-specific areas and with CoT), high-quality training data (with tool use for OOD prompts), retrieval mechanism, leveraging applications that use build-in features in their system like automatically calculated “relevancy” scores for retrieved publications and prompt engineering including CoT. As the field of AI advances, more intelligent systems beyond or including GPTs will perform more reasoning-like processes in the form of active inference rather than static inference (that can reduce “intelligence” to mere “memorization”). A new architecture that does not rely on scale (seeing more tokenized data, for instance) could potentially deliver more “intelligent” responses. The landscape of limitations and vulnerabilities evolves in tandem, requiring strategies to mitigate AI hallucinations and reduce security exploits. Guided use of GPTs can increase accuracy and reliability in the system’s results and minimize cost of use. Within the domain of scientific research, identification and supervision of the proper AI tool for tasks like retrieving and summarizing grounded peer-reviewed scientific literature is essential for continued responsible adoption of the latest advancements in AI, bringing higher alignment with human intention and trust.

## Figures and Tables

**Table 1:** Rubric for evaluating ChatGPT-4-provided articles.

Criterion	0—Not at All	1—Very Poor	2—Poor	3—Fair	4—Good	5—Excellent
Summaries of articles provided by ChatGPT-4 accurately reflect the source material.	Summary contains no accurate elements from the source	Summary is highly inaccurate, misrepresents key findings	Summary has major inaccuracies and omits key points	Summary partially accurate but missing several important details	Summary mostly accurate with only minor omissions or errors	Summary fully accurate, clearly represents source material with no errors
Articles directly address the specific research topic.	Article is unrelated to the research topic	Article is minimally related, highly off-topic	Article is tangentially related with little relevance	Article moderately related but lacks direct focus on topic	Article is relevant and addresses most aspects of topic	Article is fully relevant and directly addresses all aspects of the topic
Articles are listed in PubMed.	Not listed in any database	Not listed in PubMed	Incorrect listing in PubMed	Listed but with incorrect citation or incomplete metadata	Correctly listed in PubMed but with minor citation inconsistencies	Fully and correctly listed in PubMed with accurate citation
Articles are found on publisher’s website.	Not found on any website	Not found on publisher’s website	Incorrect listing on publisher’s website	Found but behind paywall without citation details	Found with correct citation but missing some metadata	Fully available on publisher’s site with complete citation and metadata

**Table 2:** Results of rubric scoring.

Criterion	Mean ± SD	Median (IQR)	% Scoring ≥ 4	% Scoring 5
Accuracy of summaries	4.2 ± 0.9	4 (1–5)	82%	51%
Relevance to topic	4.6 ± 0.7	5 (2–5)	89%	77%
PubMed listing	4.9 ± 0.8	5 (0–5)	96%	96%
Publisher website availability	4.9 ± 0.4	5 (2–5)	98%	98%

IQR = interquartile range; SD = standard deviation.

## Supplementary Materials

The following supporting information can be downloaded at: https://www.mdpi.com/article/10.3390/jcm14176011/s1, Table S1: Artificial intelligence software for summarizing scientific reports.
