# Supplementary material for: Pulmonary and Immune Dysfunction in Pediatric Long COVID: A Case Study Evaluating the Utility of ChatGPT-4 for Analyzing Scientific Articles
Source: J Clin Med. 2025 Aug 25;14(17):6011. doi: 10.3390/jcm14176011 (PMC12428973; doi:10.3390/jcm14176011)
Supplement: Supplementary file 1 [file jcm-14-06011-s001.zip › jcm-3683644-supplementary.pdf]

Supplemental Materials

The incidence of AI hallucinations is common pattern occurring to varying degrees. For example, as of January 2025, Elicit generally recommends approximately 90% of user-received information should be assumed accurate, balancing cost, accuracy and maximal information retrieval. Currently there is no generalizable method that matches or supersedes human performance with reliable accuracy. The suite of OpenAI GPT tools and o1 and other current frontier AI models like xAI's Grok 3, Meta's Llama 3.3, Anthropic's Claude 3.7 Sonnet and recently DeepSeek's DeepSeek-R1 have exponentially enhanced performance in tasks like program synthesis and article summarization, and numerous models are accessible through applications with easy-to-use web interfaces, API endpoints and sometimes portions of source code like open-weights for on-premise development and deployment.

Supplementary Table S1. Artificial intelligence software for summarizing scientific reports.

Features	Perplexity	Consensus	Semantic Scholar	Elicit	SciSpace	Pubtator 3.0	Research Rabbit
LLM	Default fine-tuned "Perplexity" likely using OpenAI base model, and other latest models (GPT-4o, Claude 3.5 Sonnet)	Fine-tuned "Consensus" model likely using OpenAI base model	GPT3.5-turbo-16k and/or other LLMs by Ai2	Fine-tuned GPT-3 (2022)	Fine-tuned "SciSpace" model likely using OpenAI base model	Undisclosed. Possibly fine-tuned generative AI for NER	Undisclosed
Workflow	General (can set to "Academic")	Academic	Academic	Academic	Academic	Academic	Academic
Chatbot interface	Available as searchbar	Available as separate chatbot interface integrated with OpenAI website	Available as Ask This Paper	Available as Chat with Paper	Available as Chat with Paper, Chat with PDF, available as separate chatbot interface integrated with OpenAI website	No	No
Can be accessible via OpenAI's chatbot interface	No	Yes	No	No	Yes	No	No
Copilot for paper questions	No	Yes	Yes	Yes	Yes	No	No
Search engine database	Likely Internet and/or in-house database (if academic mode selected, retrieves from academic databases like Semantic scholar)	Semantic Scholar (in 2025, 200M+ research papers)	In-house database (in 2025, 200M+ research papers)	Semantic Scholar (minimal threshold at 125 million+ research papers)	Undisclosed (200M + papers)	Undisclosed (~36M PubMed-related publications)	Undisclosed
PubMed retrieval	Yes (if academic mode is selected)	Yes	Yes	Yes	Yes	Yes	N/A
Citation included in response	Yes	Yes	Yes	Yes	Yes	Yes	Yes
Allows uploaded documents	Yes (at search bar)	Yes (as Chatbot interface)	No	Yes	Yes (can upload multiple papers at once)	No	No
Generative features	Includes search result summary, Pages	Summary of n top search results, Copilot response; Ask about this paper	Ask This Paper output, Topics in search results page	Abstract summary, methodology, limitations, etc.	Insights (from top n papers or indiividually), abstract summary, methodology, limitations, etc.	Undisclosed (can integrate with ChatGPT-4 for improved performance)	Undisclosed
Safety features/disclosures	Can turn off AI data retention	"More Results" at end of search results page specifies potentially less relevance; can identify articles from "Rigorous Journal"; beta features (like Summary) provide top 10 papers on website; includes best practices for searching and crafting queries	Disclosure of potential inaccuracy. Can request correction for a search result inaccuracy. Can opt out of data retention.	Extensive guidelines including approximated amount of sub-optimal results; there's high accuracy 1 high-accuracy column per table in results paied require	Academic AI Detector	Disclosure of potential inaccuracy	Undisclosed
Individual paper summary/pseudo-summary	If uploaded and prompted	Key Takeaway; Ask This Paper available via Semantic Scholar link	Ask This Paper	Abstact summary; Summary	Abstract summary, summary	No	No
Synthesized summary of multiple related papers	No	Top 10 papers	No	Top 4-8 papers	Top 5-10 papers	No	No
Integration with Zotero	No	Yes	Yes	Yes	Yes	No	Yes
Integration with EndNote	No	No	Yes	Yes	Yes	No	Yes
Pro benefits	Can choose from a set of advanced models	Unlimited latest model use, Copilot use, etc.	Free	Greater but limited allowance of searches, Chatbot, etc.	Advanced model, unlimited Copilot messages	Free	Free

Supplementary Table S1. Currently, there are LLM-based software with similar and unique features for literature review like Consensus, Elicit, SciSpace, Semantic Scholar, and Research Rabbit and general-purpose search like Perplexity.

Given factors like the generative nature of these large language models, large scale training data and parameterization, often involving

tens of thousands of tokens and hundreds of billions of parameters, respectively, and reduced transparency intrinsic to neural networks relative to traditional machine learning, the instance of generating false or misleading, often confident knowledge (i.e., AI hallucinations) are known to occur and remain unresolved (Jiacheng Ye, 2022; Maynez et al., 2020; Wiegreffe et al., 2022). Solutions in the form of systems or techniques are an active area of research and are being developed to mitigate the presence of AI hallucinations to enhance accuracy of model responses and reliability in AI. Evaluations by human experts in a relevant domain is still the primary method for accurate AI-generated information and instilling confidence in the system. As there are no standardized framework for identifying, quantifying, and reducing this phenomenon, it is beneficial to build an intuitive understanding of intrinsic limitations of GPTs to identify weaknesses and security vulnerabilities. Understanding transformer architecture will help to identify the most efficient and accurate LLM or LLM-based application for a specific task.

S.1. Transformer Basis of LLMs

A LLM is a neural network that is commonly built using blocks of a transformer, an architecture allowing high parallelization for efficient training and inference and flexible learning. Specifically, the transformer consists of sublayers, such as the attention layer and multilayer perceptron. In contrast with its recurrent predecessors in the field of NLP, transformers coupled with immense data and GPU/TPU hardware are more powerful, training on full sequences or contexts in parallel to capture long-range dependency between tokens. One type of transformer-based architecture is known as a GPT, as well as underlying models like GPT-4 and its improved fine-tuned variants ChatGPT-4 and 4o. A GPT has a modified attention mechanism, enabling causal masking of tokens during both training and testing. From a learning perspective, as autoregressive models, GPTs are trained to predict the next token in a sequence (i.e., unidirectionally from left to right) (OpenAI, 2023). In contrast, non-autoregressive models like Bidirectional Encoder Representations from Transformers (BERT) perform deep contextual understanding through bidirectional (from both left and right) token prediction, performing masked language modeling and next sentence prediction during pretraining (Jacob Devlin, 2018). GPTs excel in generating text and acting as chat bots, and BERT-style models achieve high performance on understanding tasks like ranking documents by relevance and sentiment analysis (Brown et al., 2023; Nogueira & Cho, 2019). Both methods of learning involve grasping context in different ways, influencing the functionality of the models.

S.2. Development from Pre-Training to Post-Training

Base models are configured from pre-training and can undergo post-training. Base models, such as GPT-4 and Llama 3, closed- and open-weights, respectively, are trained broadly and can undergo further specialization. The training data is tokenized based on a tokenization strategy (commonly byte-pair encoding or WordPiece tokenization), converting it to integers for modeling. Autoregressively, the GPT model generates a reply one token at a time based on the previous context (to the left of and up to the last generated token) and sampling of computed probabilities, tethering it to the statistical

landscape of what it has seen in training. That statistical landscape consists of language regularities like syntax and semantics found in the textual pretraining data, contained in the numerical value of the weights. For instance, if a training dataset contains sufficient and varied examples of the suffix unsigned long long (ULL) including in English text and programming contexts, the model processes and thus learns different uses and ways the suffix can relate to other words in a sequence processed in parallel (e.g., being a suffix in NULL in English and R code, and on its own ULL can refer to the unsigned long long 64-bit integer in C++).

Current forms of post-training include supervised fine-tuning (SFT), reinforcement learning from human feedback (RLHF), classical reinforcement learning (RL) or a hybrid supervised and RL approach. An SFT model like ChatGPT-4o are specifically designed with greater capability for generating conversation when prompted in text and can reduce training cost. It has been fine-tuned using a Q/A dataset to generate coherent, contextually relevant text responses, building off of its general knowledge from pretraining on Internet-derived data. Often RLHF is applied on a SFT model. Unlike RL, RLHF directly involves humans and require human annotated ranking of model responses, which in turn are used to train another neural network referred to as a reward model (RM). The RM then guides the fine-tuned LLM to generate responses aligned with a reward signal. In RLHF, humans infuse preference into the model through response selection and subsequently influence the reinforced behavior. On the contrary, as with AlphaGo, RL precluding human feedback involves the model gaming itself, training via self-play with continuous, dynamic environment interactions, optimizing its policy to behave in a way that maximizes the reward signal. In the case of AlphaGo, the reward signal was defined by a reward function—winning the game—leading to superhuman performance. A model fine-tuned with SFT and RLHF or RL such as OpenAI's o1 and DeepSeek's DeepSeek-R1 undergoes supervised learning on additional data followed by reinforcement learning to enforce greater alignment with the specified reward signal (e.g., reward signals can be given to responses that fit predefined categories of "Helpful" or "Harmless"). In the case of DeepSeek-R1, the use of SFT and RL and other techniques led to high scores on reasoning benchmarks.

S.3. The Effect of Parameterization

At a high-level, a GPT is a mathematical function that is defined by learned weights (i.e., parameters) and hyperparameters such as context window length and vocabulary size. The weights are the differentiable internals of the network, updated through gradient-based optimization (i.e., backpropagation and gradient descent), capturing knowledge conferred by training. They are often listed in versions of models to showcase scale like the 8-billion parameter model Meta-Llama-3-8B. As matrices of floating-point values, model weights are trained to transform the numerical input tokens, directly shaping the final output whether it's estimating the potential efficacy of a vaccine over another or assisting in a diagnostic prediction.

Additional factors for model performance include architectural hyperparameters like the context window length and vocabulary size and are often scaled up along with the number of parameters. The greater the length of the context window, assuming that it is accurate

and relevant, the higher the number of tokens that are readily accessible to the model for simultaneous processing and prediction. Vocabulary size represents the total possible set of tokens the model can predict for and generate. However, scaling these hyperparameters up does not always invite improvement. Models like Meta's LLaMa 65B and Google DeepMind's Chinchilla (70B), both with a context window of vocabulary size of 32,000 tokens, performed superior to OpenAI's ChatGPT-3 (175B) with a vocabulary size of 50,257, largely due to the two models being trained on significantly more data, approximately observing 4x more language usage at approximately 1.4 trillion tokens (Hoffmann et al., 2022; Brown et al., 2020; Touvron et al., 2023).

With the aforementioned tokenized, large training dataset consisting of many diverse examples of English text and code, a vast vocabulary influences tokenization granularity. A sufficiently large vocabulary would preserve, rather than fragment, the ULL suffix as a distinct token; therefore, given a large enough context window, each sequence that fits within this window that includes ULL would likely be surrounded by similar tokens—enough to show a contextual pattern and clear statistical correlation (e.g., the model finds ULL has a specific meaning in C++ across many examples). Ideally, after training and hyperparameter tuning, the tokens in the model's embedding layer (i.e., vocabulary) are representative of training data and were exposed to sufficiently sized, accurate and relevant contexts (i.e., context window) from a large, diverse, and high-quality training dataset, and subsequently the weights have learned relationships with each token in the vocabulary that, given a specific prompt, can understand and generate text effectively. With its learned parameters and configured hyperparameters, the GPT is fixed and can infer on unseen data.

When given a test dataset containing batches of sequences, the trained GPT employs sampling of a generated probabilistic distribution in the final stage of inference to determine the next predicted token. This distribution of tokens, spanning only the token available in its predefined vocabulary, is generated within the confines of what relationships it has seen in the training data (stored in the "weights"), the user's prompt and the built-up response of the model up to and including the last predicted token (fit under the "context window"). Therefore, the next predicted token in the sequence will typically be the token in the vocabulary with the highest calculated probability, assuming greedy sampling which is configurable using the test-time hyperparameter "temperature", and be contextually aligned with or similar enough to previously seen data. Both parameters and architectural hyperparameters are designed during pretraining and are set when the model's output matches patterns seen in the training dataset. In a sense, models considered open-weights are analogous to traditional compiled binaries in that their weights are like software that is downloadable and executable but remain unmappable to a human readable representation, making them effectively irreversible.

S.4. Deployment of LLMs

Since the release of the transformer paper by Google in 2017 (Vaswani et al., 2017), there is a large, diverse range of models available for deployment. LLM providers like Anthropic, OpenAI, Google, xAI, Perplexity AI and Meta offer a range of LLMs that differ in licensing terms, source code availability and where it can be accessed and deployed. On-premise deployment with sufficient hardware like on-

premise GPU clusters can be possible with downloadable weights. Remote deployment can be via a web-based AI chatbot or an API endpoint, hosted either on the company's own cloud infrastructure or an external provider's cloud platform. Open-weights base models like Meta's Llama 3.1 405B and Mistral AI's Mixtral-8x7B-v0.1 or assistants (i.e., "tuned") fine-tuned for instruction (e.g., Llama 3.1 405B Instruct, Llama 3.3-70B-Instruct, and Mixtral-8x7B-Instruct-v0.1 and Meta's Llama 3.3-70B-Instruct) are available as downloadable weights on Hugging Face as well as API endpoints on Amazon Web Services (AWS) Bedrock. Proprietary fine-tuned models like OpenAI's ChatGPT-4o and Anthropic's Claude-3.5 Sonnet are accessible exclusively through a web interface or API endpoints on external cloud platforms Microsoft Azure AI Foundry and AWS Bedrock, respectively, precluding installation while providing the infrastructure for interacting, deploying and fine-tuning these generative models. On the same web interface used by OpenAI, applications like Consensus and SciSpace are available, utilizing under the hood a LLM model that is likely by the LLM provider OpenAI with additional fine-tuning tailored for literature-related tasks (e.g., their specialized GPT could be retrieving information to augment prompts exclusively from academic databases like PubMed) (Roy et al., 2024).

S.5. Mitigating AI Hallucinations

These factual-sounding, erroneous (i.e., false or misleading) statements in many ways exhibit the intrinsic mechanics of the LLM system. Model weights are influenced by the quality of the training data. High-quality training data is diverse and representative (e.g., there are various gene expression profiles for numerous cell types under an organism, tissue, disease, and other covariates of interest with no missing meaningful type), accurate (e.g., the cellular gene expression data was not influenced by batch effect), and large (e.g., there is no predominant cell type or each cell type has sufficient examples). Post-training has evidence of increasing performance on specialized tasks (Jason Wei, 2022; Victor Sanh, 2021). By being fine-tuned on specialized data like peer-reviewed research publications, a model like those offered by Consensus and Elicit gain exposure to examples otherwise rare or absent, consisting of relevant statistical patterns in a target domain. This enriched exposure is reflected in updated weights, sealing in knowledge for future, domain-specific prompts. Currently, missing data likely includes paywalled journal publications due to current policies, omitting their findings from model training and retrieval when using web search like with search-based LLMs Perplexity and Consensus. For Out-of-Distribution (OOD) prompts, models can be trained with SFT to respond with uncertainty to questions not in the training set (Grattafiori et al., 2024).

A common limitation of many LLMs is their limited character-level processing due in part to their tokenization. The popular tokenizer BPE segments text into tokens that represent words or chunks of words, often rendering the model "blind" to finer character-level understanding that is not represented by a distinct token. This limitation can propagate into inference, where a model like ChatGPT-4o may generate the wrong count if asked the number of instances of a specific character such as the letter "C" in a long string. For example, when using the online tokenizer cl100k_base, the words "Covid" and "COVID" are tokenized differently, splitting into two tokens "C" and

"ovid," and remaining as a single token "COVID", respectively (https://tiktokenizer.vercel.app/; OpenAI Platform 2025). As compensation, a model can be trained to use tools such as a calculator, code like a python interpreter (e.g., model writes program, and another part of the system runs the program in an interpreter and passes the result back to the model), and web search for answering some prompts that are prone to hallucinations depending on its training. For instance, ChatGPT-4o could be fine-tuned using thousands of labeled examples that show the protocol for when and how to use tools, involving special tokens and decision mechanisms. When met with a likely scenario for a tool (e.g., perhaps words in the prompt like "Please use tools" or "Please use web search"), the model predicts a special token triggering the system to follow the protocol for the tool. In fact, if instructed to "Use code," ChatGPT-4o and many other models can successfully count a string of characters.

Augmenting large language models (decoder-only like GPT, encoder-decoder like BART, encoder-only like BERT) with a retrieval-augmented generation (RAG) mechanism have been adopted for various tasks like question-answering to improve performance with factual responses (Aleksandra Piktus, 2021; Fangyuan Xu, 2023; Gautier Izacard, 2021; Junxian He, 2021; Kelvin Guu, 2020; Urvashi Khandelwal, 2019). For example: the GPT PaLM-Coder with 540B, fine-tuned on the base model PaLM, led to greater performance on coding datasets relative to base models (Aakanksha Chowdhery, 2022). However, on another dataset containing student-written C-code, PaLM-Coder predicted code that likely was not to generalize to other scenarios, leaving room for future systems that confirm the validity of model responses. For many LLM-based systems like ChatGPT-4o and Perplexity, a LLM can take a cue like exceeding the knowledge cut-off in its pretraining data and conduct a web search, retrieving "relevant" information that is prepended and formatted with the user's original query for final inference. The web search can be performed using an external search engine API like Bing, an in-house database (especially if there's PII data), or other data sources. Perplexity appears to be a system that retrieves information at least partly from its own data source to augment prompts whereas OpenAI models often search via Bing. Currently, a method of integrating RAG with the now legacy GPT-4 model via a robust and secure API has been showcased to provide medical advice in the field of nephrology with promising results (Miao et al., 2024). Unlike typical LLMs like ChatGPT-4o, o1 and o1-Pro which selectively use tools to retrieve information in real-time, it appears most AI-based search applications like Perplexity and Consensus are trained to treat most user queries as an OOD problem, initiating the model to search their indexed databases and retrieve information (i.e., articles) ranked highly relevant and incorporate them into the model's input for processing. Currently, under the hood, Consensus' specialized, likely fine-tuned LLM calculates quality scores for its retrieved articles, where perhaps the presence of "Low confidence" labels indicates insufficient evidence in the response (i.e., a summary missing or fabricating the critical finding or sources). Applications like Semantic Scholar appear to maintain their own indexed search database of peer-reviewed, largely PubMed publications that serve as the database for many applications like Consensus and Elicit. Similar to training data, retrieval components in

an LLM-based system that have access to high-quality and fresh knowledge help steer the GPT to a more accurate prediction.

Recently, Perplexity AI performed post-training likely SFT of DeepSeek-R1 in an effort to remove censorship enforced by the original training (AI Team, Perplexity, 2025). Prior to Perplexity AI's fine-tuning, DeepSeek-R1 did not answer a direct question on the economic impact of a Taiwan independence on the chip company Nvidia and discussed more China and its political party, with similar behavior to other direct questions. After post-training, Perplexity AI's version of DeepSeek-R1 called R1 1776, a post-trained, less potentially biased open-weights version, could now generate direct answers and is offered on Hugging Face. Currently, there is potential for fine-tuning with RLHF system in the realm of summarization (Nisan Stiennon, 2022). For the task of summarization and citation, this kind of system could involve assigning labels based on the model's initial responses, and those responses that pass specific tests are introduced back into the chain as synthetic data where the model can learn ground truth. Applications of RL for GPTs are currently being explored with positive results (Gao et al., 2023; Long Ouyang, 2022; Yuntao Bai, 2022). With future use of RL, the reward objective must be unambiguous, unable to be broken or gamed such that the model aligns with a clear goal and produces predictable outcomes. Defining such a function is challenging in open, non-game-like environments such as generating accurate, reliable and concise literature summaries, identifying study limitations, optimizing vaccine design, or performing microbiome classification.

Given an off-the-shelf GPT like ChatGPT-4, 4o or Claude 3.7 Sonnet, optimizing input through prompt engineering can greatly enhance performance. This technique involves writing prompts that compensate for the model's limitations and are tailored to its strengths. Instructive prompts are already likely provided in the context window—invisible to the user—in AI-based applications like Consensus, Elicit and Perplexity, guiding article summarization and citation. When interacting with an application's Chat With Paper feature, it is likely the source is included in the prompt to force the model to stay within a context when answer a related question (e.g., "What are limitations in this study?" Additionally, if multi-step or complex reasoning is required when summarizing academic articles, a technique called chain-of-thought (CoT) prompting has been shown to be significantly effective with systems like GPTs (Jason Wei (2022). This process entails the generation of additional tokens that can simultaneously reveal the model's processing or "internal monologue" as well as provide richer context for the model to predict the next token. The presence of curated "cold-start" SFT data consisting of long CoT training data in DeepSeek's DeepSeek-R1 contributed to its greater stability in its subsequent RL stage relative to DeepSeek-R1-Zero which lacked this "cold start" (DeepSeek-AI, 2025). Often beneficial for multi-step, complex tasks or mathematical tasks, CoT is a focus in post-training especially with RLHF and RL and is a common behavior in OpenAI's o1, o1-Pro, DeepSeek-R1 and many other reasoning models.

S.6. Security and Ethnical Considerations

The LLM stack can be vulnerable to malicious acts. Several security attacks include prompt injections (manipulating prompts to give overriding, ulterior instructions), jailbreak attacks (bypassing security barriers through a vulnerability so the model generates

prohibited output), and data poisoning (undesirable, false information inserted in the training data that affects the output). These attacks have roots in the nature of the model architecture and the broader technology stack. Similar to traditional executables, open-weights models must have the file containing their weights downloaded in trusted, secure environments. Environments that follow certain security protocols include cloud platforms like Hugging Face, Microsoft Azure, Google Cloud Platform, AWS where there are many security barriers that monitor components of the system including network connection, API requests, etc. This can help mitigate prompt injections where user queries are altered for malice, possibly in the retrieval of additional data for the prompt that may be a fraudulent webpage (Greshake et al., 2023). Using models like the chatbot DeepSeek, whether via their web interface or the vendor's API endpoints, may involve leaking potentially sensitive data (e.g., PII) to servers located in jurisdictions where cloud compliance frameworks and privacy regulations such as HIPAA, likely differ (Khurpa et al., 2025). To mitigate security risks, deploying DeepSeek-R1 via services like AWS Bedrock can generally ensure all interactions occur through AWS infrastructure, leveraging its security and compliance features. Attacks like jailbreaking have been shown to occur when a model learns biased or adversarial patterns during pretraining or receives an adversarial input during inference (Zou et al., 2023; Qi et al., 2023). When post-training for instruction with a supervised dataset, the data can be designed to contain "poisonous" pairings (e.g., "James Bond" occurring in various contexts), causing the model to learn a kind of trigger word that, when mentioned later, can lead to harm with greater occurrence at increased model scale (Wan et al., 2023). In the medical domain, a recent publication demonstrated the possibility for large-scale data poisoning by injecting hidden medical inaccuracies in HTML documents for pretraining, finding these models passed common medical LLM benchmarks (Alber et al., 2025). Open-source models, where the complete source code including training data, model weights, scripts for preprocessing, pre-training and post-training are accessible for reproduction, appear to be uncommon (Team OLMo, et al., 2024). Prior to using any model for critical tasks, prerequisite testing should be completed that verifies the model is secure on multiple levels in order to protect the user's application. Overall, as AI innovations grow, the security landscape will continue to shift and require a kind of full-stack security framework, covering all potential attack surfaces, across the entire pipeline from training data to inference.
